# Value of one additional injection at the root of the limb in the lymphoscintigraphic evaluation and management of primary and secondary lower-limb lymphedemas

**DOI:** 10.1371/journal.pone.0253900

**Published:** 2021-07-09

**Authors:** Pierre Bourgeois, Olivier Leduc

**Affiliations:** 1 Services of Nuclear Medicine, Institute Jules Bordet and HIS-IZZ Hospitals, Université Libre de Bruxelles, Brussels, Belgium; 2 Service of Dermatology, Hospital Erasme, Université Libre de Bruxelles, Brussels, Belgium; 3 Multi-disciplinary Clinic of Lymphology, Institute Jules Bordet and Hospital Erasme, Université Libre de Bruxelles, Brussels, Belgium; 4 Service of Vascular Surgery, HIS-IZZ Hospitals, Université Libre de Bruxelles, Brussels, Belgium; 5 Department of Occupational and Environmental Physiology, Lympho-phlebology Unit, Haute Ecole HE2B ISEK, Université Libre de Bruxelles, Brussels, Belgium; University Magna Graecia of Catanzaro, ITALY

## Abstract

**Introduction:**

The classical lymphoscintigraphic investigations of lower-limb lymphatic edema [LLLE] sometimes reveal either no or few lymph nodes [LNs] at the root of the limb[s] and/or in the abdomen. The aim of the present paper is to report the results of performing one additional injection at the root of the edematous limb[s] to force the visualization of the LNs and/or to demonstrate the collateral lymphatic pathways in such patients.

**Methods and findings:**

We retrospectively reviewed our database and found 99 patients [44 primary LLLE with 47 limbs injected and 55 with LLLE secondary to treatments for cancer with 64 limbs injected] where such an additional injection had been performed.

In the 43 LLLE patients where no LNs were seen at the end of the classical exam [15 primary LLLE and 28 secondary LLLE], the extra injection showed lymphatic drainage toward LN[s] in all except 3 and when at least one LN was seen, the injection showed lymphatic drainage in every case toward the same ipsilateral [inguinal and/or iliac] LNs [as shown by the classical injection] and/or toward additional LNs.

In 40.7% of patients, we observed one or more additional lymphatic pathways: prepubic superficial lymphatic vessels [LV] crossing the midline anteriorly toward contralateral inguinal LNs in 21 [18.9%], “posterior” LV [toward contralateral inguinal LNs and/or ipsi- or contralateral lumbo-aortic and/or para-renal LNs] in 14 [12.6%], but deep LV toward the ipsilateral common iliac LNs passing between the gluteal muscles in 32 [28.8%].

**Conclusion:**

Our work pinpoints one limitation of classical bipedal radionuclide lymphangiography. In patients with primary and secondary LLLE where inguinal and/or iliac LNs cannot be seen on bipedal radionuclide lymphangiography, this additional injection reveals the true lympho-nodal status and shows unexpected collateral lymphatic pathways in 40% of cases. Such information is of the utmost importance in LLLE management and its acquisition is consequently recommended in these patients.

## Introduction

Lymphoscintigraphy, the radionuclide imaging of the lymphatic system, is now the most common way to investigate edema affecting the lower limbs [1–3]. Lower-limb edema is defined as “lymphedema” when functional and/or morphological abnormalities can be demonstrated [1,4,5]. Lower limb lymphedema [LLLE] can be either secondary [after surgery, radiotherapy, or trauma] or, less frequently, primary [2,3]. Such edemas represent a chronic [life-long] disease and can sometimes be disabling and/or severe [e.g., “elephantiasis”; lymphatic reflux in the genital organs; lymphatic leakage; when it leads to infections, etc.]. In these situations, lymphoscintigraphic images, which are usually obtained after radiocolloid injection[s] in the feet, sometimes fail to reveal the inguinal and/or iliac and lumbo-aortic lymph nodes [LNs]. Failure to visualize these LNs can, either reflect their actual absence because they have been, for example, resected in secondary lymphedema, or be a manifestation of primary lymphedema [“lymphadenoaplasia” and “hypoplasia” according to Kinmonth] [6]. However, these LNs, although present, will also not be visualized if the ability of the lower-limb lymphatic vessels [LVs] to transport the radiocolloids from the feet toward and into these nodes is so diminished that the scintigraphic tracer does not reach them and/or because progressive sclerosis has occurred [7]. Additionally, collateral lymphatic pathways may appear to overcome the “blockade” of lymphatic return in the systemic circulation [8]. These collaterals may sometimes be seen but their presence may also be underestimated due to the same limitations as previously mentioned. The aim of the present paper is to report our analysis of such situations and, in patients who were referred for the investigation of LLLE and who did not show any [and/or a reduced number of] LNs in the inguinal and/or intraabdominal areas, the results of one additional injection performed at the root of the edematous limb[s] to “force” visualization of the LNs and/or to demonstrate the collateral lymphatic pathways.

## Materials and methods

In this retrospective single-center observational review of our database, 99 patients who received such additional injection from 2008 to 2019 in the Service of Nuclear Medicine of the Jules Bordet Institute were found [see Table 1 for flow of patient selection] by the 1st author [PB] [The additional injection was systematically proposed to patients who were referred for the lymphoscintigraphic evaluation of lower limb edema and for whom the exam showed no LNs in the inguinal and/or intraabdominal areas and was performed after their informed consent]. All medical data and information regarding the patients included in this study were used in agreement with the rules of conduct dictated by the institution and in agreement with the Ethics Committee of the Jules Bordet Institute [Ethics Committee approval number 2048: patients’ informed consent is not requested for retrospective reviews and every patient is informed that their data can be used for such retrospective review].

**Table 1:** Flow chart of selection of patients for this retrospective study.

	Patients referred for investigation of LLE from 2008 to 2019	
	n = 907	
	with final diagnosis of	
1ary LLLE		2ary LLLE
n = 231		n = 237
	with no LNs or decreased number of LNs in the inguinal and/or ilio-lumbo-aortic area[s] */**	
n = 92*		n = 169**
	with our additional injection performed at the root of the limb after informed consent	
n = 44		n = 55

*/** The injection was not proposed to 18 patients with 1ary LLLE and to 22 patients with 2ary LLLE] when planar and/or SPECT-CT imaging showed LNs in nonclassical anatomical areas suggestive of the natural development of collateral pathways.

### Lymphoscintigraphic technique

Our method [9] was as follows: 0.2 mL of 99mTc-labeled [74–111 MBq per injection] human serum albumin nanocolloid [0.05 mg per injection; Nanocoll, GE Healthcare] was injected subcutaneously [using a tuberculin syringe] in the first interdigital space of each foot, with the patient supine on the exam table. The patient was instructed not to move the toes or feet during the following 30 minutes. One image of the injected site was obtained directly after the injection. The camera head was then moved to the inguinal area, where dynamic imaging was performed once to record the time to inguinal node appearance and to evaluate possible asymmetry in the nodal accumulation of the tracer. Next, one first set of whole-body images [a whole-body scan or WBS; anterior and posterior views] was obtained [“phase 1”]. For “phase 2”, one dynamic scan [lasting 15 minutes] of the inguinal area was performed but with the patient tip-toeing [flexing the toes and feet] during the sixth to tenth minute of the acquisition [with the patient still lying on the exam table and with the same goals as for phase 1, but with increased flow in the LVs of the limbs], and another set of whole-body images [anterior and posterior views] was obtained. Next [“phase 3”], a 3rd WBS was obtained after 1 hour of walking and the injected site was imaged [to calculate the percentage of radiocolloid extracted by the lymphatic system at the end of the investigation]. The whole protocol with the 3 phases [see Figs 4, 5A and 9] was completed in 3–4 hours in order to only take half of the patient’s day.

When imaging after “phase 3” showed no LNs in the inguinal and/or intraabdominal areas, another injection and additional imaging were performed [after obtaining informed consent from the patient], either on the same day as the investigation of the lower limbs or [see Figs 3, 5 and 6] on another day [according to the patient’s availability and the availability of the camera].

At this time, 0.4 mL of radiocolloid [74–111 MBq] was injected intradermally in the lateral part of the thigh in front of the greater trochanter [when this injection was performed on the same day as the 1st investigation, radiocolloid from the same vial was used for the interdigital injection; when the injection was performed another day, one-fifth of the standard Nanocoll preparation was used]. Images [anterior and posterior views, each 1–2 minutes long] were obtained after this injection, as follows. First, one or two images were obtained directly and without additional intervention to show the spontaneous lymphatic drainage of the injected area. Second, one or two images were obtained after stretching the injected site [to force the entry of the colloid into the lymphatic system]. Third, one or two images were obtained after massage movements above and limited to the injection site. Finally, images were obtained after more specific lymphatic drainage manipulation of the root of the limb and of the LVs and LNs shown in the previous images. This massage was designed to push the radiocolloid in the direction of all possible and well-known collateral pathways. With additional experience, these last massage movements were also performed with the patient lying in the lateral decubitus position. This additional investigation [“phase 4”] lasted 20 to 40 minutes at most. Single-photon emission computed tomography [SPECT-CT] of the abdomen and pelvis [see Fig 5B] was also proposed to patients and performed when the gamma camera system was available [the added value of these SPECT-CT acquisitions in these patients will be presented in another paper].

## Results

### Population

During the observational period, 301 patients out of the 907 who were referred for one lymphoscintigraphic evaluation of lower-limb edema [110 out of the 231 with primary LLLE and 191 out of the 237 with secondary LLLE] met the inclusion criteria [no LNs or decreased number of LNs in the inguinal and/or iliolumboaortic area[s]], and 99 patients among these 301 patients received the additional injection [see Table 1]. The injection was not proposed to 40 of these 301 patients [18 with primary LLLE and 22 with secondary LLLE] when planar and/or SPECT-CT imaging showed LNs in nonclassical anatomical areas suggestive of the natural development of collateral pathways.

Forty-four patients [see Table 2] had primary LLLE [31 women and 13 men; mean age, 45.5 years; range, 12–80 years]. These cases of edema were classified as “early onset” [first symptoms before the age of 35 years] in 26 patients and as “late onset” [first symptoms after the age of 35 years] in 18. In this group, 47 limbs were evaluated [2 patients had 2 exams, and 1 received injections in two limbs]. Edema was left-sided in 13 and right-sided in 19 patients. When the edema was bilateral, it was more severe on the right side in 11 patients and more severe on the left side in 4. In 15 of these limbs [31.9%], including those in 7 early-onset [26.9% of the early-onset cases] and 8 late-onset [44.4% of the late-onset cases] cases, no LNs were observed by our classical three-phase scintigraphy method.

**Table 2:** Patients characteristics.

	1ary LLLE	2ary LLLE
N patients	44	55
Women/Men	31/13	45/10
Mean age	45,5 years	56,1 years
Range	13–88	28–75
Limbs with additional injection	47	64
With NO lymph nodes seen	15	28

Fifty-five patients [see Table 2: 45 women and 10 men; mean age, 56.1 years; range, 28–83] had LLLE secondary to lymphadenectomy [in all but 2] and/or radiotherapy [in 17] for cancer [lymphoma = 2; melanoma = 15; Merkel cell carcinoma = 1; ovarian carcinoma = 3; prostate carcinoma = 3; sarcoma = 8; cervical and/or corpus uteri carcinoma = 21; vulvar carcinoma = 2]. In this group, 64 limbs [one patient underwent 3 exams, and both limbs were studied in 5 cases] were investigated, among which no LNs were observed in 28 [43.7%] by our three-phase imaging method. Edema was left-sided in 24 and right-sided in 21 patients. When edema was bilateral, it was more severe on the right side in 12 and on the left side in 7 patients.

### Lymphoscintigraphic results

#### The results can be summarized as follows:

**Fig 1:**
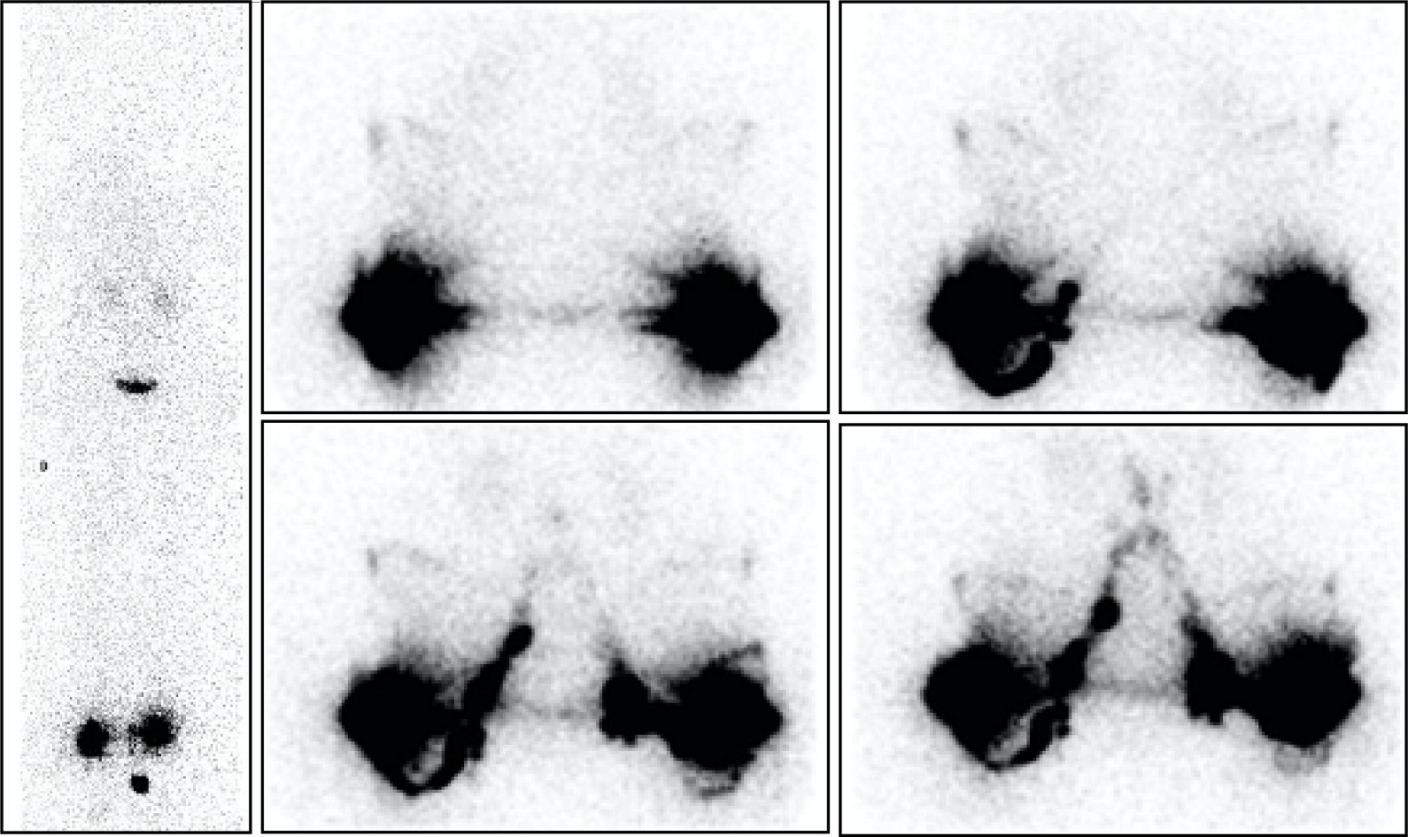
“No LNs observed in one case of primary bilateral LLLE? They are present!”. Phase 3 lymphoscintigraphy [on the left: Anterior WBS] in a 70-year-old woman with primary left-sided LLLE for 30 years showed no lymphatic vascular drainage and no infra-diaphragmatic LNs on either side. However, intradermal injections performed on the same day in the external part of the buttocks revealed LVs coursing in the direction of the right inguinal area, reaching the inguinocrural LNs and thereafter the iliac and lumbo-aortic LNs, bilaterally [on the right: Four consecutive anterior views, from left to right and top to bottom, centered on the pelvis and abdomen].

**Fig 2:**
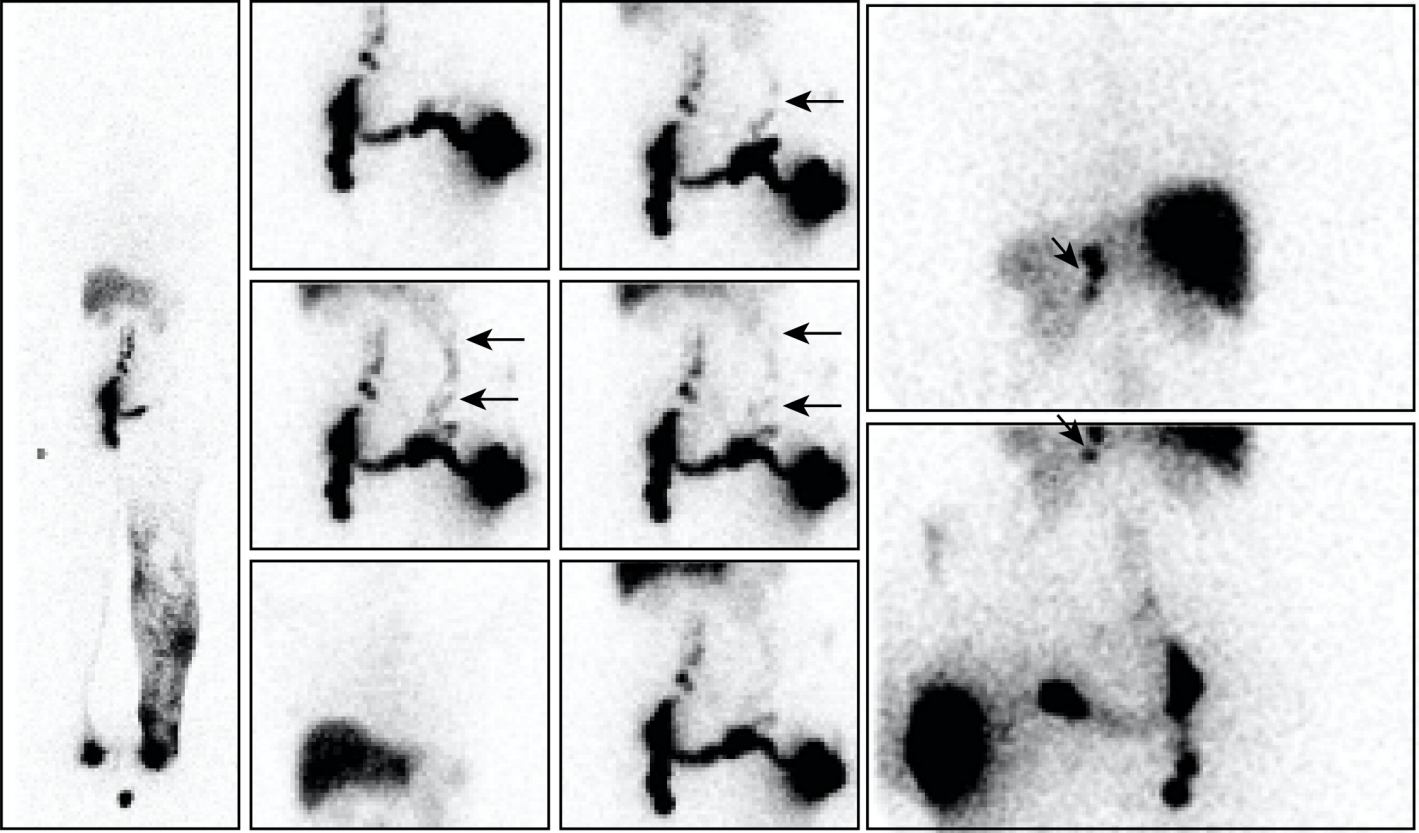
“No infra-diaphragmatic LNs in a case of primary LLLE? Inguino-crural LNs and one collateral vessel are present!”. Phase 3 lymphoscintigraphy in a 31-year-old woman with left-sided primary LLLE showed normal lymphatic drainage and normal infra-diaphragmatic LNs on the right side but lymphatic drainage from the foot to the mid-thigh through the superficial dermal lymphatic network and no infra-diaphragmatic LNs on the left side [see WBS on the left side]. The consecutive anterior views from top to bottom and right to left centered on the pelvis and abdomen, and, on the right, the posterior view [upper image] centered at the level of the liver and spleen and the anterior view centered on the pelvis and abdomen [lower image] obtained after the intradermal injection performed on the same day in the external part of the left buttock revealed LVs flowing in the direction of the right inguinal area, reaching inguinocrural LNs and, from these LNs, LVs flowing up in the left flank [horizontal arrows] to reach three left-sided lumbo-aortic LNs [oblique arrows] between the upper part of the kidney and the vertebral column.

**Fig 3:**
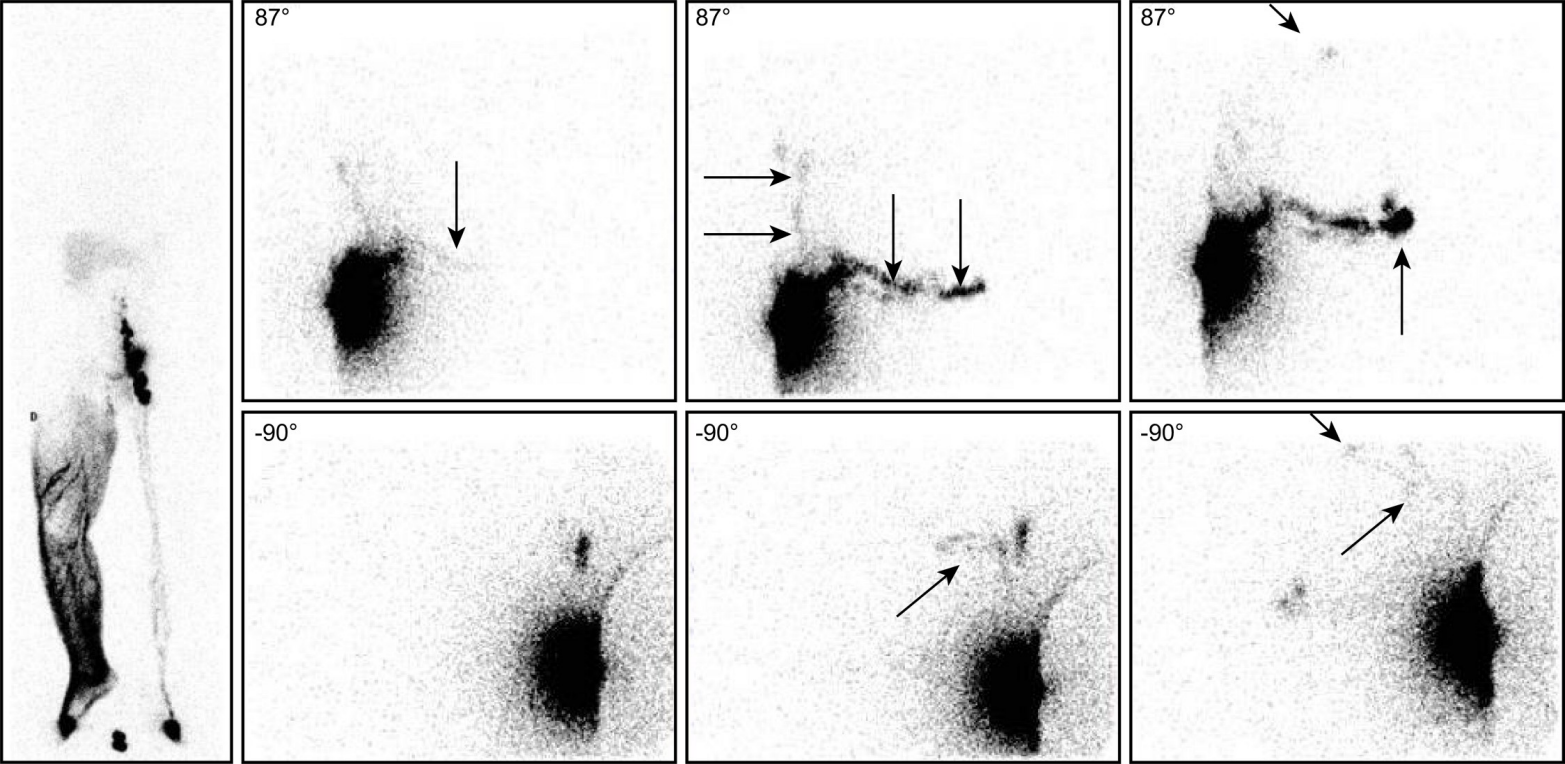
“Our injection shows collateral vessels flowing toward contralateral inguinal LNs and ipsilateral lumbo-aortic LNs”. Phase 3 lymphoscintigraphy in a 25-year-old man with right-sided primary LLLE at the level of the ankle and calf showed [see WBS on the left] normal lymphatic drainage and normal infra-diaphragmatic LNs on the left side but lymphatic drainage from the foot to the root of the limb through LVs and the superficial dermal lymphatic network on the right side. The consecutive images [on the right: From right to left, consecutive images, upper panel = anterior views; lower panel = posterior views; centered on the pelvis and abdomen] obtained after the intradermal injection performed several days later in the external part of the right buttock revealed LVs [vertical arrows from top to bottom] flowing in the direction of the right inguinal area, crossing the midline before the pubic area and reaching contralateral inguinal nodes [arrows from bottom to top] as well as LVs flowing up laterally [horizontal arrows from left to right] and then posteriorly in the left costo-lumbar area and crossing the midline [oblique arrows from bottom to top and left to right] to reach one right-sided lumbo-aortic LN [oblique arrows from top to bottom and left to right].

**Fig 4:**
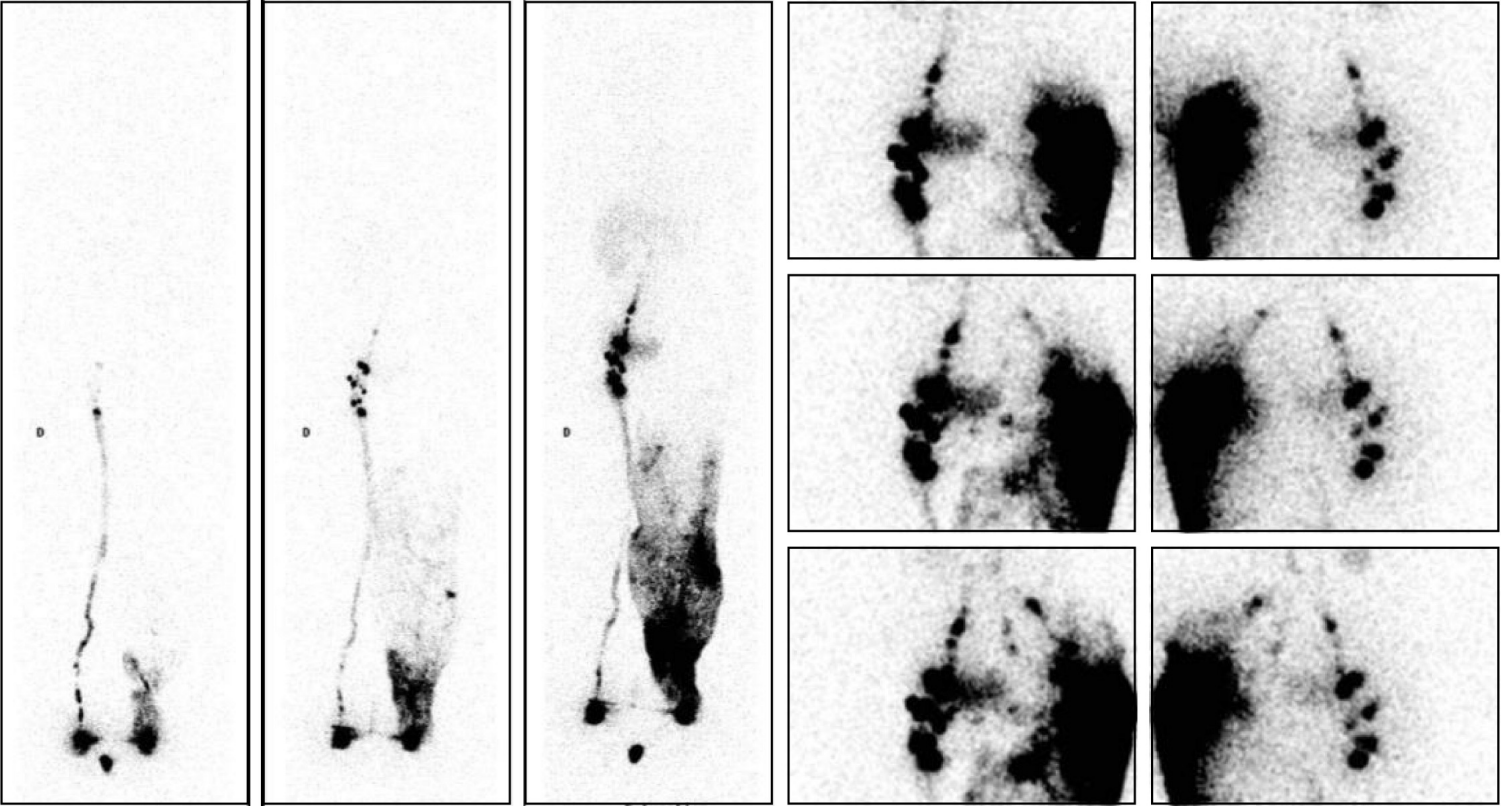
“No infra-diaphragmatic LNs in a case of left-sided secondary LLLE? Intra-abdominal LNs and collateral vessels are present!”. Lymphoscintigraphic imaging [left: From left to right, anterior WBSs obtained after 30 minutes without movement [phase 1], after five minutes of “tip-toeing” [phase 2] and after one hour of walking [phase 3] at the end of our exam] revealed in a 44-year-old woman with left-sided LLLE secondary to inguinal and iliac LN dissection, radiotherapy and chemotherapy for melanoma the following:
On the phase 1 WBS, normal lymphatic drainage from the foot to the first inguinal LN was observed on the right side, whereas progression of the tracer in one LV and in the superficial lymphatic collateral network was observed limited to the distal part of the left limb.On the phase 2 WBS, increased activity in the right inguinal LN and the appearance of an iliac LN was observed, while on the left side, progression of the tracer in the superficial lymphatic collateral network was observed up to above the knee.On the phase 3 WBS, normal lymphoscintigraphic findings were observed on the right side and on the left side, with activity in the superficial lymphatic collateral network [dermal backflow] flowing up to the mid-thigh but without LNs. The intradermal injection performed on the same day in the external part of the buttock revealed the following [right: From top to bottom, three sets of consecutive images centered on the pelvis and abdomen: Anterior views on the right and posterior views on the left]:
The tracer was observed to flow upward but also downward in the superficial lymphatic collateral network.One LV flowing toward the inferior part of the groin and another flowing toward one median inguinal LN were observed.One LV crossing the midline under the bladder to reach one “new” right-sided inguinal LN internally located with reference to the LN demonstrated by the right injection was observed.One LV reaching two left common iliac nodes was observed.At least two LVs flowing up obliquely and reaching a left lumboaortic LN were observed.

**Fig 5:**
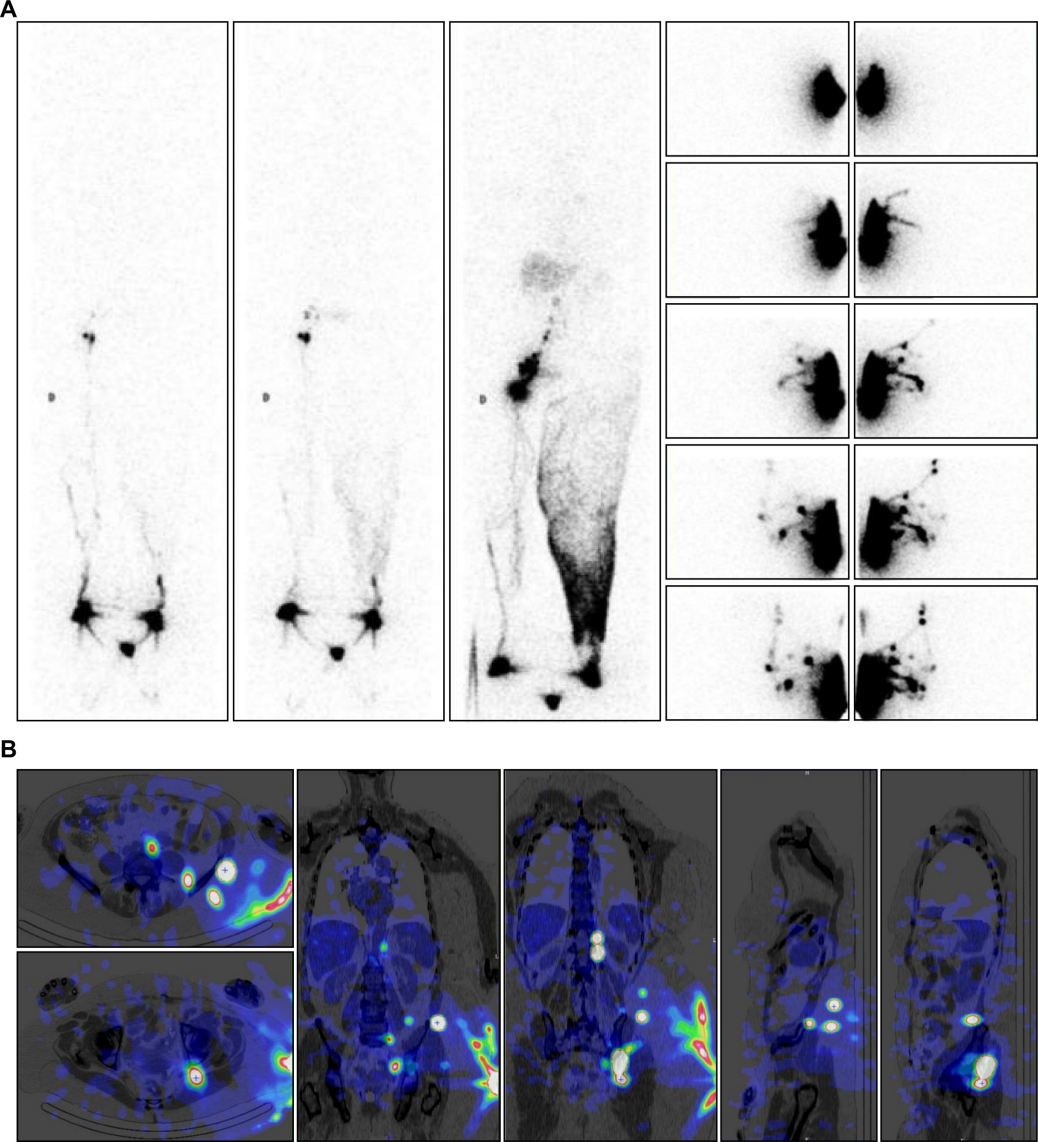
“Collateral vessels flowing toward right intra-abdominal LNs are demonstrated by the injection performed on another day!”. Lymphoscintigraphic imaging following [see Fig 5A: left: Anterior WBSs obtained [from left to right] after 30 minutes without movement [phase 1], after five minutes of “tip-toeing” [phase 2] and after one hour of walking [phase 3] at the end of our exam] in a 74-year-old woman with left-sided LLLE secondary to inguinal and iliac LN dissection for melanoma revealed in a 74-year-old woman with left-sided LLLE secondary to inguinal and iliac LN dissection for melanoma the following:
On the phase 1 WBS, normal lymphatic drainage from the foot to the first inguinal LN on the right side was observed, while progression of the tracer in one LV was observed limited to the distal part of the left limb.On the phase 2 WBS, increased activity in the right inguinal LN was observed, while on the left side, lymphatic collateral vessels were observed above the ankle.On the phase 3 WBS, normal lymphoscintigraphic findings were observed on the right side and on the left side, with activity in the superficial lymphatic collateral network [dermal backflow] from the ankle up to the groin without LNs. The intradermal injection performed several days later in the external part of the buttock revealed a complex situation with LVs reaching the following [see Fig 5A: right: From top to bottom, five sets of consecutive images [anterior views on the right and posterior views on the left] centered on the pelvis and abdomen, and Fig 5B: From left to right and top to bottom, selection of 2 transverse, 2 frontal and 2 sagittal SPECT-CT images]:
Two paravertebral LNs above the left kidney with 2 LNs intercalated “in transit” in the flank were observed.One common iliac LN was observed with one LN intercalated between the gluteal muscles and another under the osseous iliac arch.One ilio-lumbo-aortic LN was observed with two LNs intercalated at the level of the posterior part of the iliac crest.

**Fig 6:**
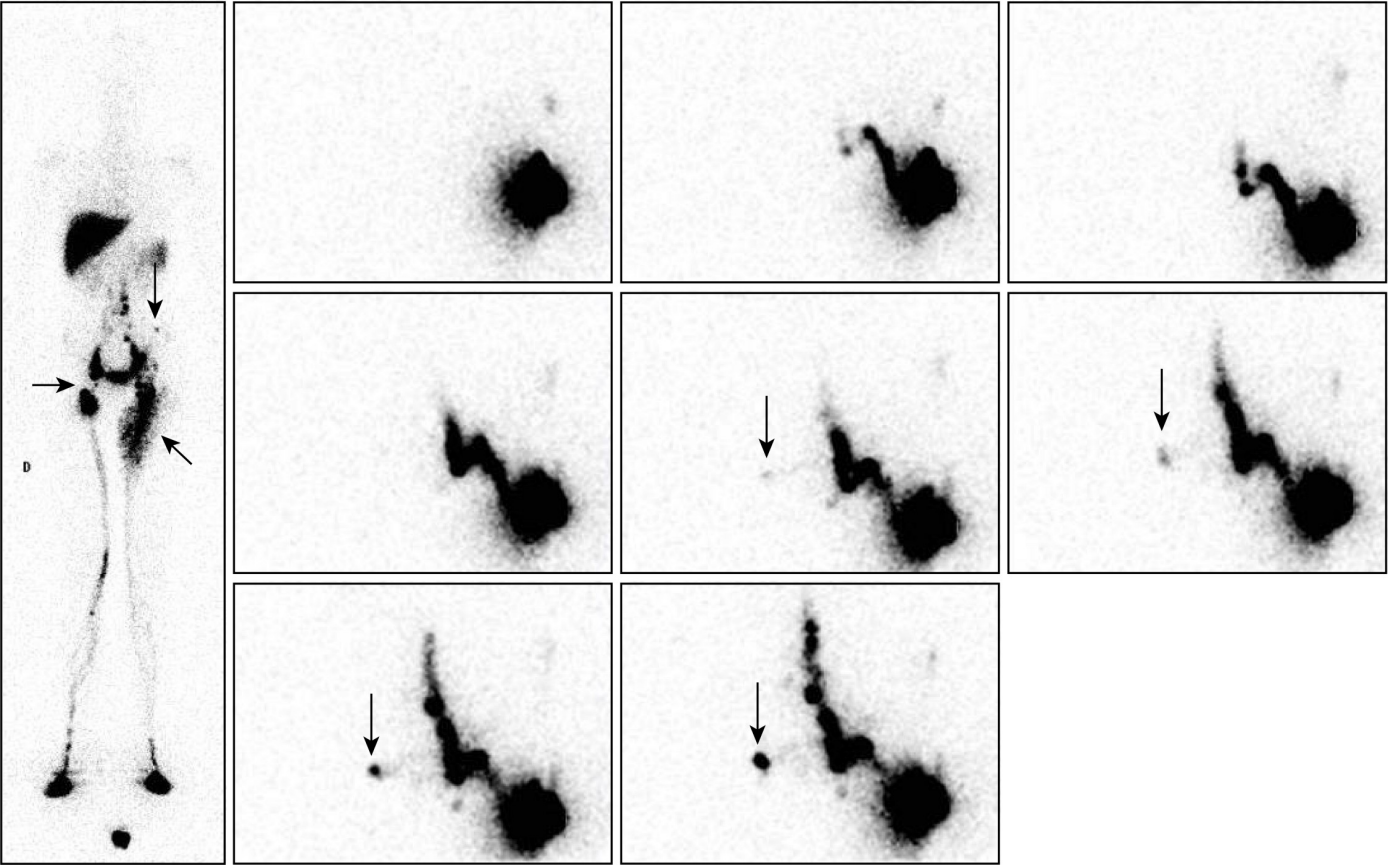
“The injection [performed on another day] shows crossed drainage from one inguinal side to the opposite side!”. Phase 3 lymphoscintigraphy [left: Anterior WBS obtained after 1 hour of walking [phase 3] at the end of our exam] revealed in a 13-year-old woman with left-sided primary LLLE at the level of the ankle and calf the following:
On the right side, normal lymphatic drainage from the foot to the root of the limb was observed, but with one inguinal nodal gap [horizontal arrow] and decreased activity in the common iliac and lumbo-aortic LNs.On the left side, normal lymphatic drainage from the foot to the root of the limb was observed, but with limited lymphatic reflux from the inguinal LNs toward the superficial dermal lymphatic network in the upper and inner third of the thigh [oblique arrow]; normal infra-diaphragmatic LN findings were also observed [“gap” in the iliac common area, irregular distribution of the inguinal LNs], but with one LN at the level of the flank [vertical arrow]. The consecutive anterior views centered on the pelvis and abdomen obtained after the intradermal injection performed several days later in the external part of the left buttock revealed [right: From top to bottom and right to left] lymphatic drainage toward the inguinocrural LNs and thereafter in the whole intraabdominal nodal axis but also one LV from the left inguinocrural LNs in the direction of the right inguinal area, crossing the midline before the pubic area and reaching one contralateral inguinal node [vertical arrows].

**Fig 7:**
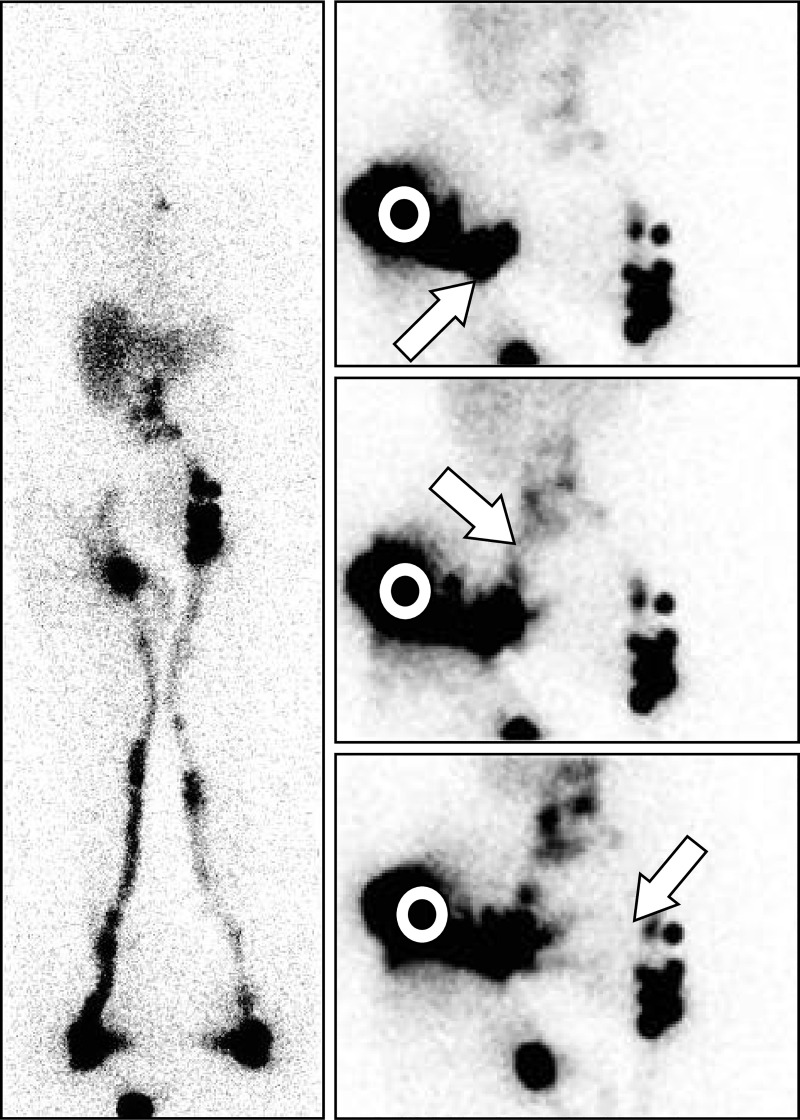
“Intraabdominal LNs and collateral vessels are shown in a case of secondary LLLE!”. Phase 3 lymphoscintigraphy [left: Anterior WBS obtained after 1 hour of walking at the end of our exam] revealed in a 69-year-old woman with left-sided LLLE secondary to surgery for inguinal leiomyosarcoma the following:
On the left side, one normal draining LV reaching one complete infradiaphragmatic LN axis was observed.On the right side, one draining LV reaching one inferior inguinal LN from which lymphatic reflux was observed with one area of faint activity in the upper inguinal area but no common iliac LNs. The three consecutive images [right: From top to bottom, anterior views] centered on the pelvis and abdomen [white circles] obtained after the intradermal injection performed on the same day in the external part of the buttock revealed draining LVs toward the inguinocrural LN [from bottom to top and left to right, oblique arrow], the common iliac LNs [from up to down and left to right, arrow] and toward the contralateral inguinal LNs in the prepubic area [from top to bottom and right to left, arrow]. Manual lymphatic drainage applied to the root of the right limb allowed reduction of the lymphedema from + 20.6% excess to + 4.9% excess [determined by comparison of the sum of the circumference values measured every 4 cm from the ankle to the root of the limb].

**Fig 8:**
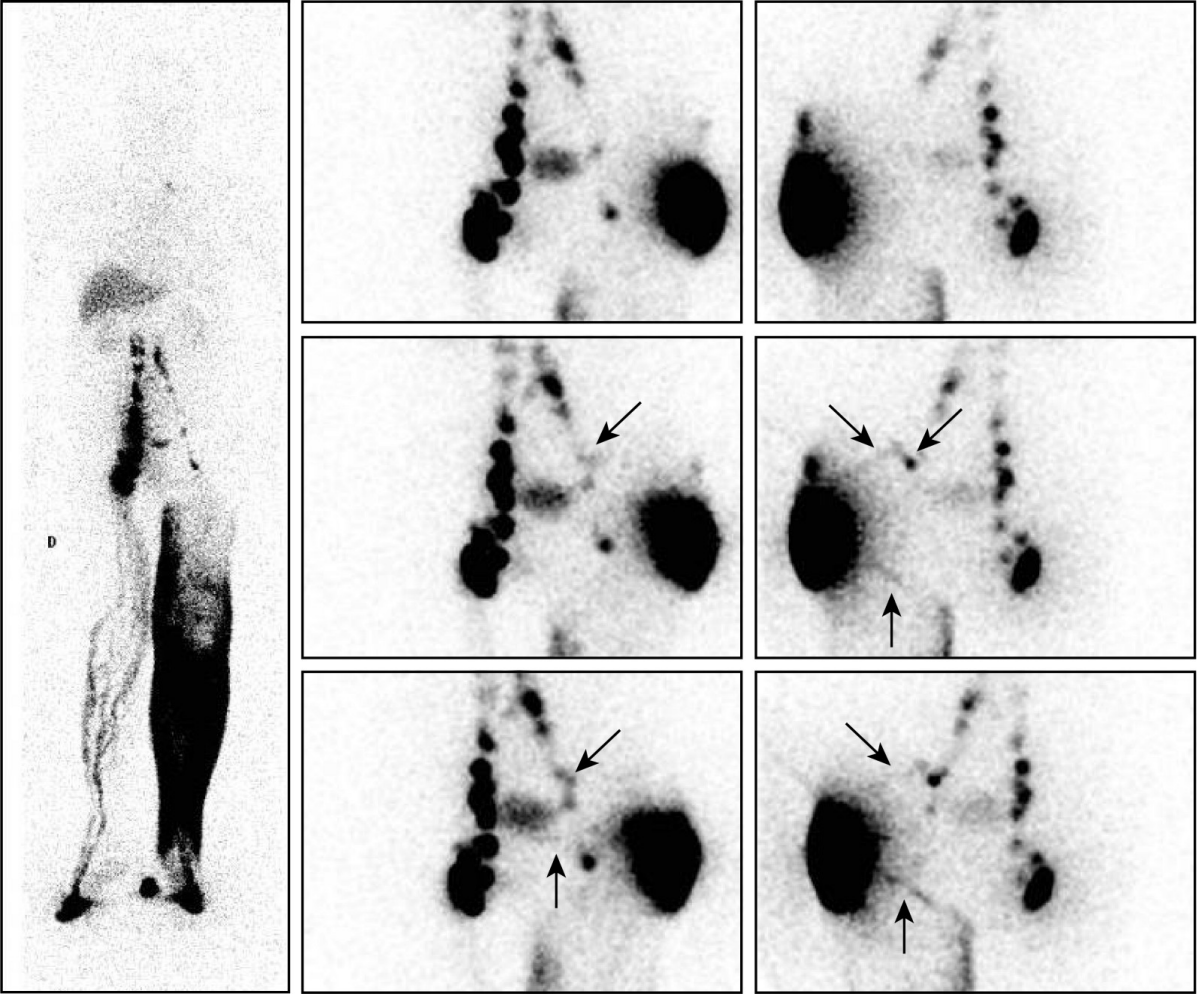
“Intra-abdominal LNs and collateral vessels are shown in a case of secondary LLLE!”. Phase 3 lymphoscintigraphy [left: Anterior WBS obtained after 1 hour of walking [phase 3] at the end of our exam] revealed in a 59-year-old man with left-sided LLLE secondary to surgery and radiotherapy for liposarcoma of the thigh the following:
On the right side, multiple draining LVs reaching one complete infra-diaphragmatic LN axis were observed.On the left side, lymphatic drainage from the ankle to the root of the limb through the superficial dermal lymphatic network was observed with one inguinal LN, one inguinocrural LN and at least one upper iliac common LN with one left-sided group of lumbo-aortic LNs [which might have received their colloidal load from the right-sided LNs]. The three sets of consecutive images [from top to bottom, anterior views on the right and posterior views on the left] centered on the pelvis and abdomen obtained after the intradermal injection performed on the same day in the external part of the buttock revealed the following:
On the posterior views, one LV flowing “up” reaching two external iliac LNs [oblique arrows] above the inguinocrural LN was observed, along with another LV flowing “down” posteriorly [vertical arrow] toward the internal part of the thigh to reach the superficial dermal lymphatic network.On the anterior views, one faintly active LV [vertical arrow] flowing toward the midline under the area of bladder activity was observed.

**Fig 9:**
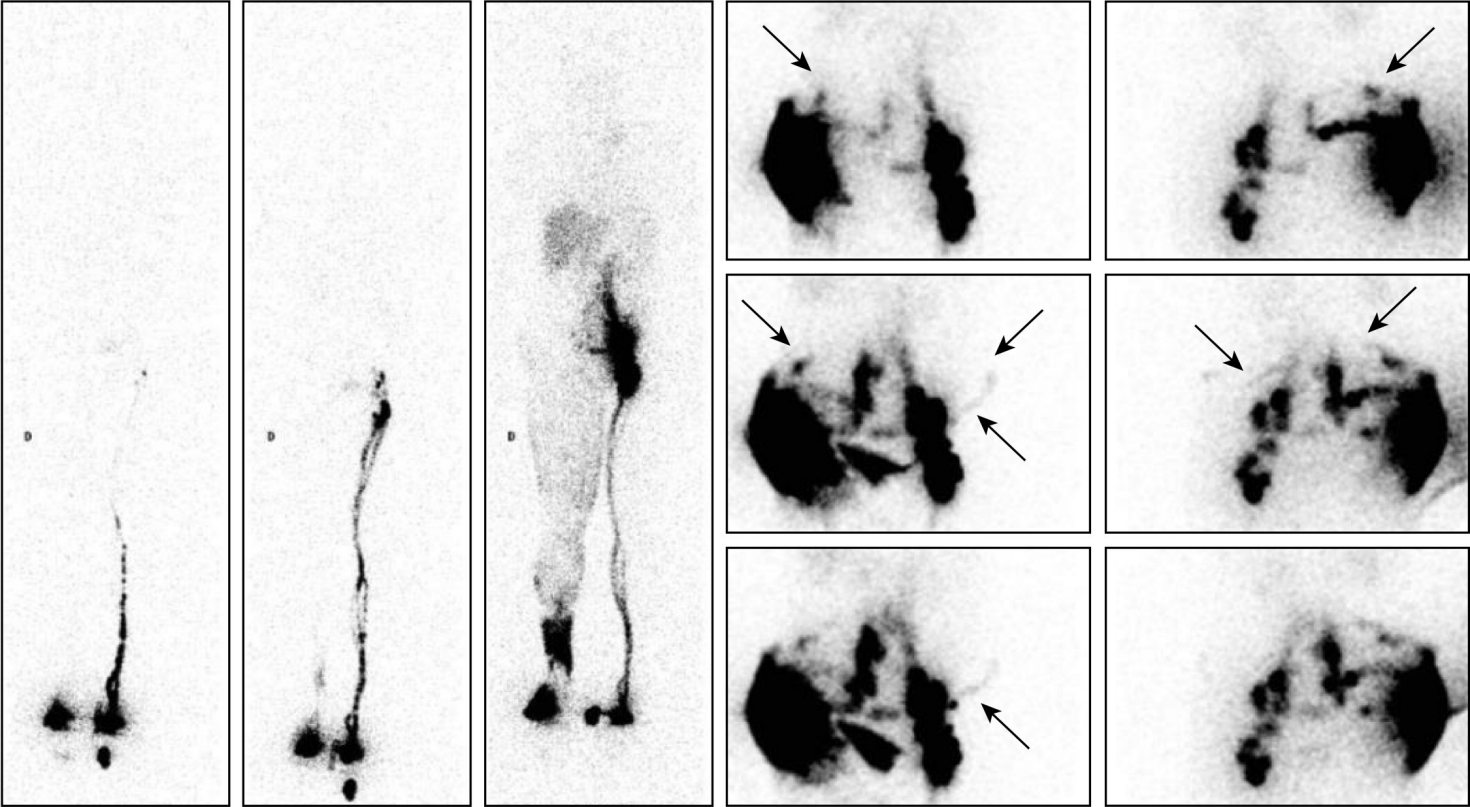
“Intra-abdominal LNs and collateral vessels are shown in a case of secondary LLLE”. Lymphoscintigraphic imaging [left: Anterior WBSs obtained [from left to right] after 30 minutes without movement [phase 1], after five minutes of “tip-toeing” [phase 2] and after one hour of walking [phase 3] at the end of our exam] revealed in a 65-year-old man with right-sided LLLE secondary to inguinal LN dissection and radiotherapy for Merkel cell carcinoma at the level of the calf the following:
On the phase 1 WBS [obtained after 30 minutes of the lower limbs remaining in a resting condition], normal lymphatic drainage from the foot to the first inguinal LN on the left side was observed, whereas no clear lymphatic outflow was observed at the level of the right foot.On the phase 2 WBS, increased activity in the left inguinal LN was observed, but on the right side, only lymphatic drainage limited to the distal part of the limb was observed.On the phase 3 WBS, normal lymphoscintigraphic findings were observed on the left side, but on the right side, dermal backflow at the level of the distal part of the foot and at the level of the ankle with faint activity in the superficial lymphatic collateral network [dermal backflow] up to the root of the limb was observed, where at least 3 LNs were visible with one iliac strain of activity. The three sets of consecutive images [right: From top to bottom, anterior views on the right and posterior views on the left] centered on the pelvis and abdomen and obtained after the intradermal injection performed on the same day in the external part of the right buttock revealed the following:
On the posterior views, three draining LVs were observed, the most important [median] reaching the right iliac LN, one [lower] flowing toward one right inguinal LN and one [oblique arrows] flowing “up” laterally then horizontally toward and crossing the midline to “come back” anteriorly in one external right-sided inguinal LN.On the anterior view, one draining LV was observed crossing the midline under the area of bladder activity toward the left inguinal LN.

- After this additional injection at the level of the LLLE for which **no LNs were observed** [see Figs 1–5], LVs draining toward LNs were identified in all 15 limbs with primary LLLE [7 “early-onset” and 8 “late-onset” cases] and in all but 3 of the 28 limbs with secondary LLLE.
- When at least one LN was observed after our classical investigation [see Figs 6–9], lymphatic drainage toward the same LN [see Figs 6 and 7] and/or toward additional LNs [see Figs 7–9] was observed in all cases.
- In cases of primary LLLE, we identified 16 lymphatic drainage vessels **different from the superficial drainage vessels directed toward the ipsilateral inguinal LN area**. They were deep [intergluteal] in 7 [14.9%] patients, crossed the midline anteriorly [see Figs 3 and 6] in 6 [12.8%] and were “posterior” [see Figs 2 and 3] in 3 [6.4%].
- In cases of secondary LLLE, we identified 48 lymphatic drainage vessels **different from the superficial drainage vessels directed toward the ipsilateral inguinal LN area**. They were deep [intergluteal: see Fig 5B] in 21 [32.8%] patients, crossed the midline anteriorly [see Figs 4 and 7–9] in 15 [23.4%], were “posterior” [see Figs 8 and 9] in 11 [17.2%], and flowed toward the ipsilateral axilla in 1 [1.5%].
- These collateral pathways were thus detected twice as frequently in secondary LLLE as in primary LLLE [and significantly more frequently in 2ary LLLE than in 1ary LLLE].
- “Posterior” lymphatic drainage vessels were observed in 6 [6%] patients, toward ipsilateral lumbo-aortic LNs in 2 [see Fig 2], toward contralateral lumbo-aortic LNs in 2 [see Fig 3] and crossing the midline to reach contralateral inguinal LNs in 2 [see Fig 9].

## Discussion

The three-phase protocol for lymphoscintigraphy in LLLE was developed in the 1980s and remains our standard approach [10]. It was adopted in 2008 by the Belgian health insurance system as one of two ways to define edema of the lower limbs as lymphatic in origin and as the rationale for reimbursement for physical therapy prescribed for these patients [11]. It is also the standard protocol proposed by the Belgian Society of Nuclear Medicine for the investigation of such edematous situations [9].

In such investigations, the absence of visualization of some LNs could be normally expected in secondary LLLE [after surgical removal and/or due to fibrosis after surgery and/or radiotherapy] and in primary LLLE [in the case of lymphadeno-aplasia]. However, the following three types of lymphoscintigraphic results led us to consider the limitations of our “physiological” approach. In the first, migration of the tracer remained limited to the distal part of the limb[s] [as in Fig 1]. In the second, no LNs could be observed, although they had been observed previously. In the third, the tracer flowed up to the root of the limb[s], sometimes revealing LNs, but to a lesser extent than could be, for example, expected in cases of secondary LLLE, especially when considering the bilaterality of the lymphadenectomies performed and the number of LNs that had been surgically removed [12].

From a practical point of view, it is possible that longer periods of walking than the hour performed by these patients would have allowed us to see [if present] these LNs. However, it would have been inconvenient to lengthen the exam for the patients [some of whom were elderly and disabled by their “elephantiasic” lymphedema].

Consequently, we began to propose one additional injection at the root of the limb[s] to our patients. We were aware that one single injection could not reveal all LNs draining to the root of the edematous limb [13], but our intradermal injection in the lateral part of the thigh in front of the greater trochanter was chosen because we wished 1] to force with such intradermal injection [better than one subcutaneous one] [14] the quick demonstration a] of the presence of the most frequently expected lymphatic drainages toward unrecognized LNs at the level of the root of the limb[s] and/or in the abdomen and/or b] of possible posterior and/or lateral collateral lymphatic pathways at the level of the pelvis but also 2] to avoid or limit the possible “stardust” effect that might mask some LNs and/or draining LVs.

Practically, and in the population referred during the observational period to our service for the investigation of lower-limb edema, we were led to propose such an additional injection to 47.6% of the patients with primary LLLE and to 80.6% of the patients with secondary LLLE. The problem is thus far from neglectable.

These additional injections are also of interest because they can demonstrate superficial and sometimes deep collateral lymphatic pathways [after the distal interdigital injection at the end of our classical 3 phases, these were observed in only 40 of 301 patients, 18 with primary LLLE and 22 with secondary LLLE]. Prepubic superficial collateral lymphatic drainage toward contralateral inguinal LNs after this additional injection was one of the two types most frequently observed, occurring in 21/107 [20%] patients in the whole series. These prepubic collateral vessels were observed somewhat more frequently in secondary LLLE [15/66, 22.7%] than in primary LLLE [6/52, 11.5%] and more frequently in secondary [5/28, 17.8%] than in primary [2/17, 12%] LLLE among patients without LNs.

If these prepubic collateral vessels were expected, less expected were:

[1] the demonstration of posterior superficial collateral vessels flowing from the external injected part of the thigh toward its lateral and posterior part, the flank and the posterior costo-lumbar area to reach the intraabdominal LNs and/or crossing the midline to finally reach the contralateral inguinal LNs and

[2] deep lymphatic drainage direct to the common iliac LNs [as in Fig 5, demonstrated by SPECT-CT, with LVs passing between the gluteal muscles and sometimes with intercalated LNs].

These deep intergluteal draining LVs, running from the buttock toward the iliac common LNs are described in the anatomical atlas. To our knowledge, however, this is the first report of these intergluteal LVs as well as LVs from the external part of the buttock flowing back and crossing the posterior abdominal wall toward the contralateral inguinal LNs, as demonstrated by lymphoscintigraphy.

Strikingly, after this additional injection, we demonstrated collateral lymphatic pathways in 6/17 [35%] cases of primary LLLE and in 21/28 [75%] cases of secondary LLLE in limbs without ipsilateral LN visualization [2p < 0.01] and in 9/35 [26%] cases of primary LLLE and in 26/31 [84%] cases of secondary LLLE in limbs with ipsilateral LN visualization [2p < 0.001]. The greater number of collateral pathways identified in cases of secondary than primary LLLE may reflect the poorer development and/or function of the lymphatic vasculature in the latter.

### Practical implications

The use of this additional injection might have important implications for the management of patients with primary and secondary LLLE.

According to Kinmonth [6], who used X-ray lymphangiography, primary LLLE can be classified into three groups: distal peripheral lymphangio-dysplasia [abnormal lympho-vascular drainage but a normal LN situation], proximal lymphadeno-dysplasia [normal lympho-vascular drainage but an abnormal LN situation], and these two combined. For patients with such LLLE, the prognosis and evolution of these lymphangiographic presentations seem indeed different [15] and worse for those with proximal lymphadeno-dysplasia. With this additional injection and its result, the patient can be better informed, and his/her follow-up can be changed appropriately [with control proposed every year rather than every three years].

These results also have several practical therapeutic implications for surgeons. The demonstration of LNs at the root of the limb raises the possibility of performing LN-to-vein anastomosis [16–18] as well as, when no LNs are demonstrated at the root of the limb, the question of whether the results of one [simple] classical lymphoscintigraphic approach should be used to support LN grafting, as previously proposed [19]. The demonstration of naturally open and functioning lymphatic pathways from one inguinal area to that on the opposite side [as in Fig 3] may also raise questions about the utility of LV transplantation [20,21]. The presence of [inguinal and/or intraabdominal] LNs and/or collateral vessels, as shown with the additional injection, must at least be kept in mind when analyzing the clinical results of such interventions and their lymphoscintigraphic controls. In fact, in any study in which LN grafting is proposed at the root level of a lymphedematous limb, it should be ensured that LNs are truly absent and simply not shown by the injection in the foot.

For physical therapists [who usually work in a “blind” way on all the possible collateral lymphatic pathways they have learned] [8,22], this “phase 4” injection can reveal the collateral LVs present in their patients and which the therapist may stimulate to keep them open. Observation of the prepubic lymphatic pathways supports the physical therapy proposed for secondary LLLE. However, in primary LLLE, manual lymphatic drainage of the contralateral lymphatic pathways is not classically mentioned in the initial treatment plan [22]. Following our observations, the practice of physical therapists must adapt to the new information. The unexpected draining LVs observed during phase 4 lymphoscintigraphy should help them drain the edema, and their sessions of manual lymphatic drainage [usually limited to 30 minutes] might become more efficient and cost effective. The protocol of our exams [and the resulting images] is systematically given to the patients with our recommendations to their physical therapists to adapt their treatment to these results [when possible] and to stimulate merely the collateral vessels shown by this additional injection. To date, we only have case reports of some clinical improvement in the edema when the therapist took such information into account for patients in whom their classical “blind” approach was unsuccessful [as in Fig 7]. Regardless, the interest of such an injection for the physical therapist requires future studies comparing the results of their treatment with and without this information, as proposed in the EFforT-BCRL trial [23].

The results of the lymphoscintigraphic exam may also be relevant to the prescription of elastic stockings and the choice between garments that reach the top of the thigh and those that include the pelvis [“panty” or “hemi-panty” styles], with the goal of avoiding the compression of superficial collateral LVs draining the edematous limb. Compression garments are the most important tool for preserving and improving the therapeutic success achieved during treatment with complete decongestive therapy.

### Limitations of our work

One of the limitations of our work is that although the additional injection was systematically proposed to the patients meeting the inclusion criteria, it could not be systematically performed. The availability of the camera [and of the tracer] had to be taken into account and the availability of the patients and the reluctance of some to undergo such an additional injection of a radioactive tracer also represented a problem.

However, we think that our results are underestimated merely for the following reasons:

- The majority of images captured after these intradermal injections were only planar [anterior and posterior views], and it was sometimes difficult to say if the LNs observed were due to deep and/or superficial lymphatic drainage.
- The simultaneous injection of the radiocolloid in both feet, when leading to the unilateral visualization of LNs, may hamper the demonstration of one LN [among these “heterolateral” LNs which may be “hyperactive”] as receiving activity from our additional injection.
- Activity in the bladder may hinder the recognition of faint signals from the prepubic LVs where these vessels are in front of this area of bladder activity [see Figs 2 and 4].
- Our first investigations were also performed with the patients lying in the supine position, which may have hindered lymphatic flow in the posterior LVs because these vessels were compressed by the pressure of the patient’s weight.
- We did not always have the time needed to perform lengthy massage of all the potentially present collateral lymphatic pathways.
- Finally, our manual lymphatic massages may have been less efficient than those performed by well-trained professional physical therapists.

### For other lymphoscintigraphic approaches?

These limitations may, however, be overcome by using one or more of the following approaches [they are now routinely proposed to our patients, and they present their own advantages but also limitations and/or disadvantages]:

- In patients with unilateral LLLE, the tracer can be injected into the foot of only the edematous limb in a first attempt at visualization. With such an approach, crossing draining LVs that have spontaneously opened, even in the presence of LNs at the root of the limb, may be seen [for example, in secondary LLLE and especially in patients with clinical signs of edema at the level of the root of the limb, thumb, genital area and/or prepubic area]. Limiting the injection to only the edematous limb has the disadvantage of missing any functional and/or morphologic lymphatic abnormalities in the “normal” nonedematous contralateral limb. These cryptic or clinically latent abnormalities can be found in both primary and secondary cases [24,25].
- Patients with either unilateral or bilateral LLLE can be invited back for a second investigation with our additional injection at least several days after the first diagnostic three-phase procedure. This approach has two advantages: [1] the injection is then performed under optimal conditions for scintigraphy [see Figs 3, 5 and 6] [with the possible addition of one SPECT-CT acquisition with the advantages: see Fig 5B] [26]; and [2] this second investigation can include one or more well-trained physical therapists of the institutional department of physiotherapy [or the patient’s own physical therapist] who will then perform one session of manual lymphatic drainage to try to open the collateral pathways [probably more efficiently than the author PB could]. The disadvantage for the patients is clear: they have to visit the clinic a second time. However, in our experience, when the advantages of a second investigation are explained well, patients generally agree to one. For physical therapists, such sessions are opportunities to directly see the results of their practice.

### Imaging alternatives?

Lymphofluoroscopy, near infrared fluorescence imaging of LVs after the injection of indocyanine green, has been proposed as a nonradioactive alternative to lymphoscintigraphy [27–29]. It yields more detailed images than lymphoscintigraphy and might reveal some lymphatic pathways and/or dermal backflow phenomena better than the latter. However, it can detect only superficial fluorescent structures, not deep LNs [unlike planar lymphoscintigraphy and, even better, lympho-SPECT-CT]. Its users include merely surgeons performing lymphatico-venous anastomosis [29,30]. They appreciate its ability to show pre-operatively [and per-operatively] the LVs to be anastomosed. The technique has also been used by physical therapists [23,31]. To date, however, indocyanine green is officially approved only for imaging sentinel LNs in cancer of the cervix and corpus uteri [32], and there are concerns about its potential toxicity to LVs [33,34].

Magnetic resonance lymphangiography [MRL] is another alternative to lymphoscintigraphy [35–37] and is also largely used by surgeons [38]. They appreciate the resolution of the MRL images and the fact that they can visualize the LVs, the LNs [eventually with no colloidal uptake] and the veins surrounding the LVs for anastomosis. However, no reports of the ability of MRL to diagnose the collateral pathways shown in our study and the LNs with no colloid uptake could be found. MRL is also less available and more expensive, and its use is currently constrained due to the requirement for the off-label subcutaneous injection of gadolinium chelates [36,38]. Unless such a magnetic contrast agent is injected, the lymphatic flows and their orientations cannot be analyzed.

## Conclusion

Our work pinpoints one limitation of classical bipedal radionuclide lymphangiography. In patients with primary and secondary LLLE where inguinal and/or iliac LNs cannot be observed, the additional injection shows the true status of LNs in at least one-third and reveals the collateral lymphatic pathways in 40% of these cases. The information is of the utmost importance in LLLE management by surgeons and physical therapists, and its acquisition is consequently recommended in these patients.
